# Graph Theoretical Analysis of EEG Functional Connectivity Patterns and Fusion with Physiological Signals for Emotion Recognition

**DOI:** 10.3390/s22218198

**Published:** 2022-10-26

**Authors:** Vasileios-Rafail Xefteris, Athina Tsanousa, Nefeli Georgakopoulou, Sotiris Diplaris, Stefanos Vrochidis, Ioannis Kompatsiaris

**Affiliations:** Centre for Research and Technology Hellas, Information Technologies Institute, 6th Km Charilaou-Thermi, 57001 Thessaloniki, Greece

**Keywords:** emotion recognition, EEG, multimodal physiological signals, functional connectivity, graph theory, multimodal fusion

## Abstract

Emotion recognition is a key attribute for realizing advances in human–computer interaction, especially when using non-intrusive physiological sensors, such as electroencephalograph (EEG) and electrocardiograph. Although functional connectivity of EEG has been utilized for emotion recognition, the graph theory analysis of EEG connectivity patterns has not been adequately explored. The exploitation of brain network characteristics could provide valuable information regarding emotions, while the combination of EEG and peripheral physiological signals can reveal correlation patterns of human internal state. In this work, a graph theoretical analysis of EEG functional connectivity patterns along with fusion between EEG and peripheral physiological signals for emotion recognition has been proposed. After extracting functional connectivity from EEG signals, both global and local graph theory features are extracted. Those features are concatenated with statistical features from peripheral physiological signals and fed to different classifiers and a Convolutional Neural Network (CNN) for emotion recognition. The average accuracy on the DEAP dataset using CNN was 55.62% and 57.38% for subject-independent valence and arousal classification, respectively, and 83.94% and 83.87% for subject-dependent classification. Those scores went up to 75.44% and 78.77% for subject-independent classification and 88.27% and 90.84% for subject-dependent classification using a feature selection algorithm, exceeding the current state-of-the-art results.

## 1. Introduction

Affective computing has been a growing field of research, aiming to develop systems and devices being able to recognize, process, and simulate human emotions. Since the paper of Rosalind Picard [1] in 1995, a plethora of research has been made in the field, including applications such as healthcare [2], video games [3], product development [4], and human–computer interaction (HCI) [5]. Such systems can offer to the development of artificial intelligence since emotion recognition is a fundamental aspect of human intelligence [6].

To perform emotion recognition, it is important to understand the nature of emotions. Emotions have a variety of ways to be described depending on the culture, language, or even subject. Thus, distinguishing between emotions is a very difficult task. For this manner, lots of researchers have adopted a 2D representation of the emotions based on valence and arousal. This 2D model can offer a catholic way to describe emotions, thus making the emotion recognition task feasible. Based on this model, emotions are described by the pleasure or disliking they produce to the subject and their intensity. Valence describes the nature of the emotion, being positive or negative, while arousal describes the intensity of emotion, being weak or strong. The valence–arousal space can have either continuous representations of emotions or discrete points.

Since emotions are psychophysiological processes, there are physiological attributes capable of describing the different emotional states. The recent development of non-intrusive sensors to monitor physiological signals with minimum obtrusiveness has led to the rise of emotion-recognition applications deploying physiological sensors, such as electroencephalograph (EEG) [7], electrocardiograph (ECG) [8], galvanic skin response (GSR) [9], and more. Recognizing emotions requires the extraction of meaningful patterns from the gathered physiological data. The complex nature of emotions and their depiction on physiological signals have led to the use of multiple sensors together to improve emotion recognition performance. Multimodal emotion recognition can be complemented by the unique physiological responses that each modality provides [10].

Connectivity analysis of EEG signals has been used for emotion recognition [11,12]. Nevertheless, the use of graph measures derived from the connectivity patterns of EEG has not been adequately studied in the field of emotion recognition. EEG is known to be an indicator of various diseases, such as dementia [13], or even motor imagery [14]. Motor imagery can be considered a classification problem, therefore it can be addressed using different machine learning techniques [15]. Apart from machine learning, fusion techniques, such as majority voting [16], and optimization methods, such as genetic algorithm (GA) [17], also have been studied for such applications. Graph measures, as an analysis process of EEG signals, have also proved to be promising indexes of neurodegenerative diseases, such as Down Syndrome [18] and Alzheimer Disease [19], epilepsy [20], and other disorders [21]. They also are promising biomarkers for explaining the development of the typical behavior of infants [22]. Therefore, the possible role of these measures in the field of emotion recognition is studied in this research.

In this work, a novel framework for emotion recognition is proposed based on functional connectivity analysis of EEG signals and network science indices along with a fusion scheme of EEG and peripheral physiological signals. Functional connectivity of EEG signals is computed using Mutual Information (MI) between the electrodes. The extracted connectivity networks are further processed by computing graph theoretical measures, which describe integration and segregation characteristics of the network. The graph theory features extracted are concatenated with simple statistical features derived from peripheral physiological signals. Subject-dependent and subject-independent models for binary valence and arousal score classification were trained. The proposed framework was evaluated on the publicly available dataset DEAP [23], which includes EEG and peripheral physiological signals from 32 different subjects. Three different machine learning algorithms, namely support vector machines (SVM), Random Forest (RF), and extreme gradient boosting (XGB) decision trees, along with Convolutional Neural Network (CNN), are used for the valence and arousal classification task.

The main contributions of this work could be summarized as follows:

- Assessing the performance of graph theory analysis of EEG signals for the problem of emotion recognition.
- Proposing a novel framework for multimodal emotion recognition from EEG and peripheral physiological signals. The novelty of the method stands in the exploitation of graph theory measures for the feature extraction of EEG signals, along with a fusion scheme of these graph theory features with statistical features from peripheral physiological signals.
- Testing the accuracy of different classifiers and a CNN for the emotion recognition problem based on the aforementioned analysis framework.
- Examining the performance the proposed framework in two different scenarios; a subject-dependent scenario and a subject-independent scenario.
- Evaluating the two different scenarios of the proposed framework using the DEAP dataset [23].

The rest of the paper is organized as follows: in Section 2 the related work is presented followed by Section 3 where our proposed method is described. Section 4 presents the results of our method and compares them with other state-of-the-art methods using the DEAP dataset. Finally, in Section 5, the conclusion and future work proposals are presented.

## 2. Related Work

Before describing the methods and the results of our work, delving into the current state-of-the-art in the field of emotion recognition using physiological sensors is needed. In the following section, a review of the main methods of the current state-of-the-art is presented, separated into single modality and multimodal emotion recognition.

### 2.1. Single Modality Emotion Recognition

Over recent decades, various sensors have been deployed for emotion recognition applications. They can mainly be separated into external and internal measurements. The vast majority of applications using external measurements are based on computer vision analysis [24] and speech recognition [25]. In this context, Kar et al. [26] proposed a three stage method for facial expression recognition from facial images. Their system is based on extracting features from the facial images and then reducing their volume by applying principal component analysis (PCA) and linear discriminant analysis (LDA). The final classification was performed using an SVM classifier. In [27], authors developed an enhanced neural network architecture able to predict different emotions based on the analysis of facial expressions from videos. Zhao et al. [28] developed a complex deep learning model based on CNN to predict different emotions from speech data from two public datasets achieving results of over 90% for both datasets.

Internal measurements are derived from physiological sensors. Such sensors provide insights into the internal state of each subject. Emotion recognition applications based on physiological sensors attempt to correlate these insights with the users sentiments by proposing different methods based on the modality deployed. The physiological sensors can further be divided into EEG and peripheral signals. EEG can provide insights into brain function, which can be helpful in emotion recognition. In the work of [7], a two-channel EEG was used for emotion recognition. Fourier and wavelet-based features were extracted and fed to a gradient boosting decision tree (GBDT) classifier achieving an accuracy score of 76.34% in predicting valence. Doma et al. [29] performed multiple tests of emotion recognition using EEG data and classic machine learning algorithms on a publicly available dataset. They found that, when performing PCA and also split data into time segments, the accuracy was increased from 50–65% to 55–75% along with increases in precision and f1-score. Deep learning methods have also been utilized to analyze and perform emotion recognition using EEG signals. In [30], two different convolutional neural network techniques were used, performing accuracy scores of 61.5% and 58.01% in arousal, and 58% and 56.28% in valence estimation. In the work of Wang et al. [11], a connectivity analysis on EEG signals was performed by computing the phase-locking value (PLV) between each pair of electrodes. Then, a PLV-based graph CNN (P-GCNN) was trained for binary valence and arousal classification, achieving 84.35% classification accuracy for SEED dataset, and 73.31%, 77.03%, and 79.20% average classification accuracies for valence, arousal, and dominance classifications, respectively, on the DEAP database.

Apart from EEG, other peripheral physiological signals can also provide useful knowledge in understanding and predicting emotional states, by providing information about other vital signals, such as heart rate and respiration. Such signals include GSR, heart rate (HR), ECG, and electromyography (EMG). In [9], photoplethysmography (PPG), which provides HR data and GSR were deployed along with various feature selection and machine learning algorithms to perform three-class emotion recognition. Results indicated that GSR features were able to recognize emotions successfully with SVM classifier performing the best. ECG data along with transfer learning was used in [8]. The authors built two different networks: the first dealing with unlabeled and the second with labeled data. Their results outperformed state-of-the-art methods achieving accuracy scores of 96.3% and 96% in the SWELL dataset, and 84% and 85.8% on the AMIGOS dataset in valence and arousal, respectively. Research by using facial EMG has shown that the number of subjects influences the emotion recognition accuracy [31,32,33]. In [34], long short-term memory (LSTM) network has shown not to be influenced by the number of subjects achieving an accuracy from 92.28% for 9 emotions up to 99.09% for 2 emotions.

### 2.2. Multimodal Emotion Recognition

Apart from deploying a single modality, multimodal solutions for emotion recognition have found a lot of use. By deploying multiple sensors and combining them with the proper method the accuracy of emotion recognition can be increased, taking advantage of the unique characteristics of each modality. Simple feature level fusion techniques, such as concatenation [35], and decision level techniques [36], have been also used with reasonable results. Gong et al. [37] perform a hybrid fusion of ECG, EMG, respiratory changes (RSP), and skin conductivity (SC) taking advantage of both fusion methods. Apart from simple fusion methods, some researchers have developed more advanced methods for fusing different modalities to perform emotion recognition. In [38], the authors used the ASCERTAIN dataset consisting of EEG, GSR, ECG, and facial expression (EMO) features. The fusion method they proposed was vertex-weighted multimodal multi-task hypergraph learning which is based on hypergraph construction reaching an accuracy of 74.34% on valence and 79.46% on arousal.

Another commonly used method for multimodal emotion recognition is feature selection. When dealing with multiple modalities, the amount of features is usually quite big and also often contains redundant information. By applying feature selection techniques dimensionality reduction can be achieved retaining only the most useful of the features. In this line, such methods have been adequately studied, such as the Fisher score [39] and mutual information-based feature selection methods [40]. Torres-Valencia et al. [41] performed margin-maximizing feature elimination and recursive feature elimination based on an SVM classifier on two publicly available datasets. They found that the more relevant features were those of the EEG for emotion recognition. In [42], a feature selection method based on reinforcement learning was compared to other random selection, sequential, and genetic algorithm (GA) based feature selection methods. They found that their Interactive Feature Selection method performed better than the other feature selection methods.

The development of deep learning over recent years has led researchers to apply such methods for multimodal emotion recognition. In the work of Zhang [43], a combination of EEG and facial expression has been proposed for emotion recognition. The model is based on a decision tree and bimodal deep automatic encoder achieving an accuracy score of 85.71% on discrete emotions. A hierarchical CNN has been proposed in [44] to combine EEG and peripheral signals for emotion recognition. CNN has also been used in [45] combined with LSTM for the fusion of video and audio signals for emotion recognition. Authors in [46] present a new database for emotion recognition that includes face, body gesture, voice, and physiological signals. They also proposed different deep belief networks (DBN) with the convolutional DBN performing the best. In the work of [47], an accuracy of 89.53% was achieved using a DBN and SVM classification for the fusion of EDA, PPG, and zygomaticus EMG sensors for emotion recognition. In [48], the authors proposed a bimodal LSTM for emotion recognition based on physiological signals. They achieved 93.97% mean accuracy on the SEED dataset and 83.53% on the DEAP dataset. Wu et al. [12] proposed a method based on connectivity analysis from EEG, and the selection of critical emotion subnetworks. Classification accuracies from the fusion of the proposed EEG analysis with eye-movement analysis were 85.34 ± 2.90% and 86.61 ± 3.76% for arousal and valence on the DEAP dataset, respectively.

## 3. Materials and Methods

In this section the methodology followed in this research is described in detail. This section includes the description of the dataset and the data analysis procedure, which includes the feature extraction methods and the experimental design.

### 3.1. Dataset

**Figure 1:**
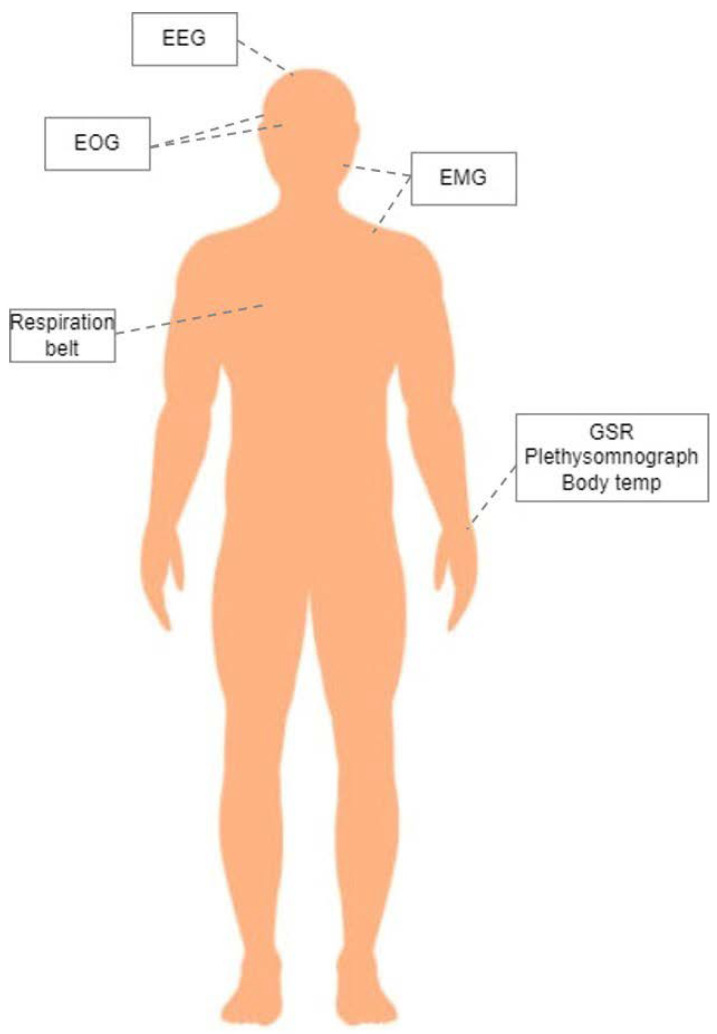
Schematic representation of the deployed sensors in the DEAP dataset and their place on the human body.

In this study, the DEAP dataset was used for multimodal emotion recognition [23]. The DEAP dataset contains EEG signals from 32 electrodes and peripheral physiological signals from 8 different sensors, those being vertical and horizontal EOG, Zygomaticus and Trapezius EMG, GSR, respiration belt, Plethysomnograph, and body temperature. These modalities are linked with different emotion responses by providing information regarding head, mouth, and eye movements; heart rate and respiration rate; and temperature and sweat gland activity [23]. A schematic representation of the different sensors deployed in the DEAP dataset and their position on the human body can be seen in Figure 1.

**Figure 2:**
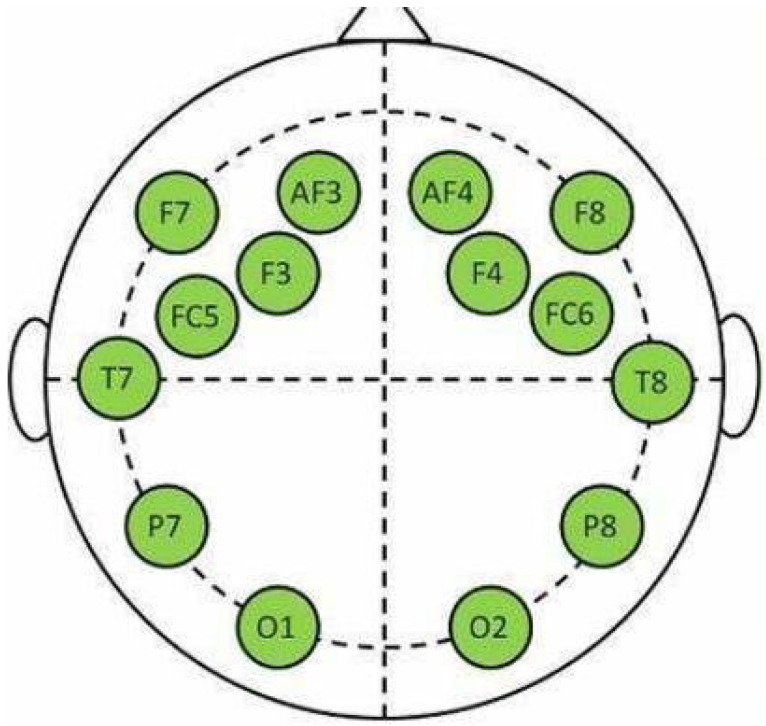
Location of the 14 channels of the Emotiv epoc+. Image from [50].

The data were collected from 32 participants using 40 different 1-minute-long video stimuli. Each participant rated each video in terms of valence, arousal, dominance, and liking. The ratings are on a scale from 1 to 9. In our study, only the valence and arousal ratings were used to perform our experiments, which are the values used for the 2D representation of emotions. Valence represents whether an emotion is positive or negative, while arousal describes its intensity [49]. The dataset provides preprocessed data, where all signals were resampled to 128 Hz. The EEG data were further preprocessed, removing EOG artifacts and passing through a 4–45 Hz bandpass filter. From the 32 EEG channels, the 14 channels included in the Emotiv epoc+ [50] were used, which can be seen in Figure 2.

### 3.2. Data Analysis

**Figure 3:**
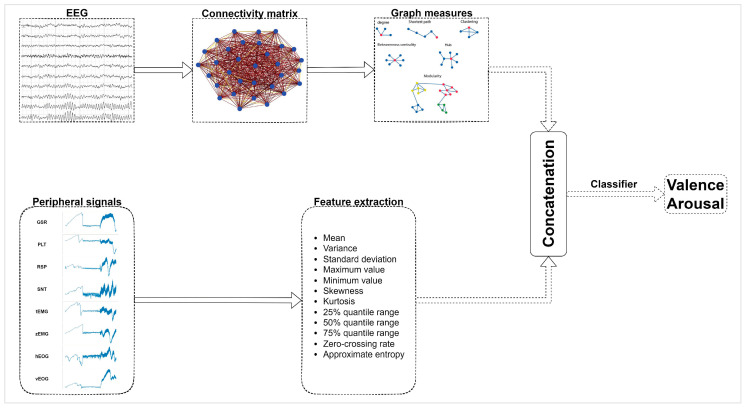
Analysis pipeline. The analysis includes statistical feature extraction from the peripheral physiological signals and graph measures extraction from the connectivity matrices of EEG signals. The features are concatenated before being fed to the classifier for the binary valence and arousal classification.

The data analysis consists of two different steps; the extraction of useful features from the EEG and peripheral physiological signals, and the train of algorithms for the classification of valence and arousal. The analysis procedure is depicted in Figure 3.

#### 3.2.1. Feature Extraction

Feature extraction from both the EEG and peripheral signals was performed using a sliding window technique. The window applied was 4 s long with a step of 2 s. The features extracted were both time and frequency domain features. The same 12 time-domain features were extracted from all peripheral physiological signals, those being mean; variance; standard deviation; max; min; skewness; kurtosis; 25%, 50%, and 75% quantile range; zero-crossing rate; and approximate entropy. These features describe basic statistical attributes of the input signals, thus giving an insight to the behavior of the signals. This results in 12 features × 8 peripheral physiological signals for a total of 96 features from peripheral physiological signals.

For the EEG-based feature extraction, a graph measure approach was applied. For each time window, at first, a connectivity analysis between the electrodes was performed. The connectivity between the electrodes was extracted by applying the mutual information algorithm. Mutual information is a non-directional connectivity measure, which reveals both linear and non-linear statistical dependencies. Because the information flow within the brain includes many highly non-linear processes, the use of mutual information can be helpful in detecting functional coupling between different brain regions [51]. The computation of mutual information between all different pairs of electrodes led to an adjacency matrix for each window. Then, graph measures of the network were computed, including global and local efficiency, transitivity, clustering coefficient, betweenness and degree centrality, characteristic path length, modularity, and density.

Characteristic path length is the average shortest path length of the edges connecting the nodes of the network [52]. Global efficiency is the average inverse shortest path length of the network [53]. Local efficiency of a node is the computation of global efficiency on a local level [53]. Transitivity of a graph is the ratio of closed triplets to the maximum number of triplets (open and closed) [54]. An open triplet is three nodes with one and/or two connections between them, while a closed triplet is three nodes with three connections between them (i.e., a triangle). The clustering coefficient of a node is ratio of its connected neighbors to the maximum number of possible connections [52]. Modularity is a measure of the degree to which the network can be divided into clearly defined modules [55]. Betweenness centrality measures the importance of a node in the communication of the network other nodes and corresponds to the fraction of all shortest path that passes through the node [54]. Degree centrality of an individual node is equal to the number of links connected to that node [54]. Finally, the density of each graph is the sum of all the weights of the graph.

All the computed features, along with their total number can be seen in Table 1. We resulted in a total of 224 features per window; 128 graph measure features, and 96 features from the peripheral physiological signals. Each window obtained the valence and arousal score of the corresponding video.

#### 3.2.2. Experimental Design

After extracting the features for all subjects, the valence and arousal scores were dichotomized into low (≤4.5) and high (>4.5) scores, to perform binary classification of the valence and arousal scores.

Our experimental design consists of testing three different feature sets to define which set of features performs the best; those feature sets being the features from peripheral physiological signals, the graph theory features extracted from the EEG signals and their fusion. For the fusion of peripheral physiological signals features and the graph theory features, concatenation was tested for feature level fusion and a feature selection method. Feature selection is a process responsible for selecting a feature subset which performs the best by reducing the input size and also removing the redundant information from the initial feature set. GA was chosen to be applied for the feature selection method.

The GA algorithm is an optimization algorithm finding application in various fields, such as machinery condition monitoring [56] and servo systems [57]. The GA algorithm is based on the natural selection and aims to maximize a fitness function. The fitness function chosen is the classification accuracy. The process of natural selection starts with the selection of the individuals performing the best from a initial population. During each iteration of the GA, which is named generation, a new population is produced from the previous selected individuals, which are called parents, through the process of crossover and mutation. Through each generation the best performing set of individuals is chosen according to the fitness function. When the total number of generations is completed, the best performing from all the chosen individuals is our final optimization solution. In the case of feature selection, the chosen individual through each generation is the feature subset that achieves the highest accuracy score.

**Figure 4:**
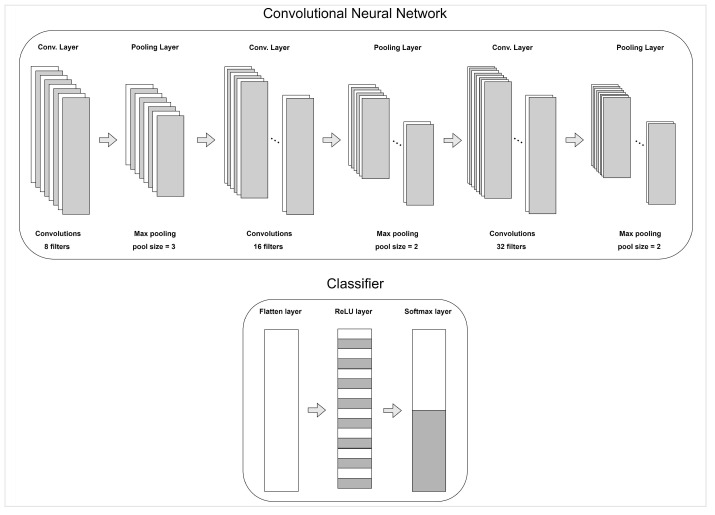
Architecture of the CNN used for the valence and arousal classification.

Three different classifiers were tested, namely RF, SVM, and XGB. We also applied a 1D-CNN fed with the features and also performed a feature selection using GA. The CNN consists of three convolutional layers each one followed by a pooling layer. The optimizer applied was the Adam optimizer and the loss function was the binary cross-entropy loss function. The architecture of the CNN can be seen in Figure 4.

Our method was tested using two different experimental framework; subject-dependent and subject-independent framework. In the subject-dependent framework the data of each subject is used to train a subject-specific model and test its performance using data of the same subject. In the subject-independent framework, the data of one subject are used as test data, and the model is trained using the data of the rest of the subjects.

## 4. Results and Discussion

In this section, the main results of both the subject-dependent and the subject-independent frameworks. A comparative analysis with other state-of-the-art methods for both frameworks is also performed, after which the results of this study are discussed.

### 4.1. Subject-Dependent Results

After extracting features from the different signals, the performance of the different feature sets was tested in the binary classification of valence and arousal. At first, the performance of each feature set was tested separately; namely the peripheral physiological signals features and the graph theory features from the EEG signals, and their fusion in a subject-dependent binary classification of valence and arousal. The extracted features of each subject were split to training and testing sets with a ratio of 85/15. The mean and standard deviation accuracy results of the binary valence and arousal subject-dependent classification across all subjects are presented in Table 2. From the results, it is clear that the concatenation of peripheral physiological signals features and the graph theory features from the EEG signals improve the classification accuracy. The best performing algorithm for the binary classification is the CNN, achieving the best performance throughout all different feature sets.

Since the best performing feature set is the concatenation of peripheral physiological signals features and graph theory features, the GA-based feature selection algorithm was applied to this feature set. For the GA parameters the number of generations was set to 200, the number of solutions per generation was set to 10, the size of each generation was set to 100, the size of pooling was set to 4, and the number of mutations was set to 3. A comparison of the results of the use of GA-based feature selection method versus the results of the whole feature set are presented in Table 3. In the table, the mean and standard deviation of the binary classification accuracy across all subjects are presented. From Table 3 it is clear that the use of GA-based feature selection improves the overall accuracy of the binary valence and arousal classification, by removing the redundant information from the feature set. The best performing algorithm is again the CNN.

In Table 4, a comparison of our work with other state-of-the-art works with subject-dependent models using the same dataset is presented. It can be seen that our method exceeds the state-of-the-art methods for both the valence and arousal score prediction.

The results of Table 4 reveal the superiority of our method compared to other state-of-the-art methods for the binary classification of valence and arousal using peripheral physiological signals and EEG signals. However, these results are based on subject-specific models, meaning that each subject has its own model which is trained only from his/her data and is specifically built for him/her. It is important to also study the performance of our method in a subject-independent scenario, where the trained model is unaware of the data of the test subject. To this line, a Leave-One-Out Cross Validation (LOOCV) was performed for all subjects, where each time the data of a specific subject was the test data and all the data of rest subjects were the train data.

### 4.2. Subject-Independent Results

Since, in all cases (with and without GA-based feature selection), the CNN was the best performing algorithm only the CNN was tested to assess its performance for the LOOCV experimental design. The performance of the whole feature set of the concatenated peripheral physiological signals features and the graph theory features was tested, along with the GA-based feature selection algorithm. For the feature selection process, the train dataset was split into an input dataset and an evaluation dataset using a 85/15 ratio. After the feature selection process was finished, the training and testing procedure was performed normally.

The mean and standard deviation accuracy results of all subjects are presented in Table 5. From the results it can be seen that the feature selection method massively increase the accuracy results to a reasonable level of 75.44% for valence and 78.76% for arousal classification.

The subject-independent models have lower performance compared to the subject-dependent models, which is in line with other works [58,59]. This result is expected since physiological signals are highly dependent on each subject. The physiological responses to a specific stimuli differ for each different subject. Thus, subject-specific models can better detect the unique physiological responses to the different emotional stimuli. Nevertheless, it is important, also, to have high-accuracy subject-independent models for emotion recognition in real life applications, where, in most of the cases, the train of a subject-specific model is not possible. Therefore, the comparison of our subject-independent model results with other state-of-the-art methods is of great importance.

In Table 6, a comparison of our method with other state-of-the-art methods having also subject-independent models and using the same dataset. From the table it can be seen that our method performs better than most of the current state-of-the-art method and has comparable results with the best performing method reported.

## 5. Conclusions

Affective computing through the analysis of physiological sensors is a fundamental aspect of the development of HCI. The exploitation of EEG and peripheral physiological signals can provide insights into human internal state, thus contributing to the task of emotion recognition. Among the most common techniques of EEG analysis is functional connectivity computation, which leads to the formation of networks between brain regions. Nevertheless, the application of network science indices calculation from these networks has not been studied in detail for emotion recognition. Even though graph theory is a known method in the analysis of EEG, its results in predicting emotional states has not been studied in depth.

In this work, a novel framework of EEG analysis and fusion with peripheral physiological signals is proposed. The novelty of the presented work lies in the exploitation of graph theory measures from EEG signals for the classification of valence and arousal. The analysis is based on the computation of EEG functional connectivity networks and the extraction of graph theory-based features from these networks. The graph theory measures are concatenated with statistical features extracted from peripheral physiological signals. Our method was tested in two different experimental frameworks, with subject-dependent and subject-independent models. Average accuracy results of the subject-dependent framework from the DEAP dataset across all subjects using a CNN were 88.27% and 90.84% for valence and arousal binary classification, respectively. These results exceed results of current state-of-the-art studies of subject-dependent models using the same dataset. The results of our subject-independent framework using GA-based feature selection and CNN for binary valence and arousal classification are 75.44% and 78.76%, respectively. These results are comparable with current state-of-the-art methods on the same dataset using subject-independent models. This study demonstrates that the use of network characteristics of functional connectivity patterns of EEG signals provides valuable information for the application of emotion recognition and that the proposed feature level fusion scheme of EEG and peripheral physiological signals represents a promising technique for the task of emotion recognition. Therefore, the beneficial role of graph theory indexes derived from connectivity analysis of EEG signals and different feature and decision level fusion techniques for the combination of EEG and peripheral physiological signals in the application of emotion recognition need to be studied.

## Figures and Tables

**Table 1:** Features computed along with their total number for each time window. The features of the peripheral physiological signals were computed once for each one of the 8 different modalities.

Peripheral Physiological Signals Features	
**Feature**	**Total Number of Features**
Mean	8
Variance	8
Standard deviation	8
Maximum value	8
Minimum value	8
Skewness	8
Kurtosis	8
25% quantile range	8
50% quantile range	8
75% quantile range	8
Zero-crossing rate	8
Approximate entropy	8
**Global Graph Measures**	
**Graph Measure**	**Total Number of Features**
Characteristic path length	1
Global efficiency	1
Transitivity	1
Modularity	1
Density	1
**Local Graph Measures**	
**Graph Measure**	**Total Number of Features**
Clustering coefficient	32
Local efficiency	32
Betweenness centrality	32
Degree centrality	32
**Total**	224

**Table 2:** Accuracy results from the experimental analysis of all subjects (mean ± standard deviation) using different feature sets. The first column pair represents the results of the peripheral physiological feature set, the second pair represents the results of the graph theory feature set and the last column pair includes the results of the concatenation of these feature sets.

	Physiological Features		Graph Theory Features		Concatenation	
	**Valence**	**Arousal**	**Valence**	**Arousal**	**Valence**	**Arousal**
**SVM**	68.5 ± 4.76	71.12 ± 6.42	71.5 ± 5.21	72.58 ± 7.12	82.4 ± 5.39	81.15 ± 8.39
**RF**	72.7 ± 5.18	73.64 ± 5.12	75.2 ± 5.19	78.27 ± 6.26	82.68 ± 5.77	81.9 ± 7.09
**XGB**	73.2 ± 4.76	75.34 ± 8.07	79.8 ± 4.98	80.12 ± 8.51	83.41 ± 6.09	82.92 ± 7.41
**CNN**	76.5 ± 5.14	78.24 ± 7.35	81.2 ± 5.41	80.89 ± 6.72	83.94 ± 6.77	83.87 ± 7.72

**Table 3:** Comparative accuracy results from the experimental analysis of all subjects (mean ± standard deviation) with and without the use of GA-based feature selection. The first two columns refer to the case where no feature selection method was implemented. The last two columns refer to the case where feature selection method was implemented.

	Without GA Feature Selection		With GA Feature Selection	
	**Valence**	**Arousal**	**Valence**	**Arousal**
**SVM**	82.4 ± 5.39	81.15 ± 8.39	85.71 ± 5.27	84.37 ± 7.32
**RF**	82.68 ± 5.77	81.9 ± 7.09	87.65 ± 4.68	86.92 ± 6.06
**XGB**	83.41 ± 6.09	82.92 ± 7.41	87.78 ± 4.99	87.72 ± 6.39
**CNN**	83.94 ± 6.77	83.87 ± 7.72	88.27 ± 5.43	90.84 ± 6.15

**Table 4:** Comparison of our accuracy results with other state-of-the-art methods using subject-dependent models. In the second column the method used for the valence and arousal classification is presented. The last two columns refer to the best accuracy results (mean±standard deviation) for the valence and arousal classification.

Paper	Method	Valence	Arousal
Wang et al. [11]	Connectivity analysis of EEG signals with PLV P-GCNN for binary valence and arousal classification	73.31 ± 11.66	77.03 ± 11.49
Tang et al. [50]	Differential entropy features from EEG signals for ϑ, α, β, and γ frequency bands Time-domain statistical features from peripheral physiological signals Bimodal-LSTM network for binary valence and arousal classification	83.82 ± 5.01	83.23 ± 2.61
Zhang et al. [44]	Statistical features from EEG and peripheral physiological signals Hierarchical features from EEG using Hierarchical CNN Weight-based feature fusion RF model for binary valence and arousal classification	84.71 ± –	83.28 ± –
Wu et al. [12]	Connectivity analysis of EEG signals with Pearson correlation Emotion-relevant critical subnetwork selection Eye-movement features Deep canonical correlation analysis model for binary valence and arousal classification	85.34 ± 2.90	86.61 ± 3.76
Our work	Connectivity analysis of EEG signals with MI Graph-theory features from EEG Statistical features from peripheral physiological signals Concatenation and feature selection with GA 1D-CNN for binary valence and arousal classification	88.27 ± 5.43	90.84 ± 6.15

**Table 5:** Comparative accuracy results of the LOOCV experimental setup with and without the use of GA-based feature selection. The results refer to the mean and standard deviation of the accuracy results across all subjects.

	Without GA Feature Selection		With GA Feature Selection	
	**Valence**	**Arousal**	**Valence**	**Arousal**
**CNN**	55.62 ± 4.42	57.38 ± 6.12	75.44 ± 5.14	78.76 ± 5.42

**Table 6:** Comparison of our accuracy results with other state-of-the-art methods using subject-independent models. In the second column, the method used for the valence and arousal classification is presented. The last two columns refer to the best accuracy results (mean ± standard deviation) for the valence and arousal classification.

Paper	Method	Valence	Arousal
Pandey et al. [60]	Variational Mode Decomposition feature extraction from EEG signal Deep neural network for binary valence and arousal classification	62.5	61.25
Chao et al. [61]	Frequency domain features from EEG signal Feature mapping using multi-band feature matrices CapsNet network for binary valence and arousal classification	66.73	68.28
Joshi et al. [62]	Power spectral density and Hjorth parameter features from EEG for ϑ, α, β, and γ frequency bands Differential entropy and Differential and rational asymmetry features from EEG Bimodal-LSTM network for binary valence and arousal classification	75.5	76
Xing et al. [63]	Stack auto-encoder decomposition method for EEG decomposition Frequency band powers from the decomposed EEG signals LSTM network for binary valence and arousal classification	81.1	74.38
Our work	Connectivity analysis of EEG signals with MI Graph-theory features from EEG Statistical features from peripheral physiological signals Concatenation and feature selection with GA 1D-CNN for binary valence and arousal classification	75.44 ± 5.14	78.76 ± 5.42

## Data Availability

The DEAP dataset analyzed in this study is available to all researchers and can be assessed upon approval. This data can be found at http://www.eecs.qmul.ac.uk/mmv/datasets/deap/index.html (accessed on 26 September 2022).

## Abbreviations

The following abbreviations are used in this manuscript:

EEG	Electroencephalograph
CNN	Convolutional neural network
HCI	Human–computer interaction
ECG	Electrocardiograph
GSR	Galvanic skin response
MI	Mutual information
SVM	Support vector machines
RF	Random forest
XGB	Extreme gradient boosting
PCA	Principal component analysis
LDA	Linear discriminant analysis
GBDT	Gradient boosting decision tree
PLV	Phase locking value
P-GCNN	PLV-based graph CNN
HR	Heart rate
EMG	Electromyograph
PPG	Photoplethysmograph
LSTM	Long short-term memory
RSP	Respiration
SC	Skin conductivity
GA	Genetic algorithm
LOOCV	Leave one out cross validation
